# Fibrinogen-to-albumin ratio and long-term mortality in oldest-old patients undergoing percutaneous coronary intervention

**DOI:** 10.1186/s12877-025-06111-4

**Published:** 2025-07-02

**Authors:** Yalin Cheng, Huimin Li, Chenguang Yang, Haiyang Gao, Peng Li, Wanrong Zhu, Yuzhu Lu, Fusui Ji, Xue Yu, Wenduo Zhang

**Affiliations:** 1 Department of Cardiology, Beijing Hospital, National Center of Gerontology, Institute of Geriatric Medicine, Chinese Academy of Medical Sciences, Beijing, 100730 China; 2 Department of Cardiology, Beijing Hospital, National Center of Gerontology, Institute of Geriatric Medicine, Chinese Academy of Medical Sciences & Peking Union Medical College, Beijing, China

**Keywords:** Coronary artery disease, Mortality, Fibrinogen, Albumin

## Abstract

**Background:**

The fibrinogen-to-albumin ratio (FAR), a novel inflammatory marker, has demonstrated prognostic utility in cardiovascular diseases. However, its role in risk stratification among oldest-old patients (≥ 80 years) undergoing percutaneous coronary intervention (PCI) remains poorly established.

**Methods:**

This single-center retrospective cohort study enrolled 641 consecutive patients aged ≥ 80 years with coronary artery disease who underwent PCI between 2015 and 2021. Based on the median FAR value (0.079), patients were divided into higher FAR and lower FAR groups. The endpoints were cardiovascular and all-cause mortality. Multivariable Cox models and restricted cubic splines assessed the associations between FAR and endpoints.

**Results:**

During a median follow-up of 61 months, 237 deaths (37%) were recorded, of which, 124 (19.3%) were due to cardiovascular disease. The 1-year mortality was 9.3% and 5-year mortality was 27.4%. Kaplan-Meier analysis demonstrated higher FAR levels were significantly associated with increased risk of both cardiovascular and all-cause mortality (log-rank *p* < 0.001). According to the restricted cubic spline, the association between FAR and mortality was J-shaped. Higher FAR (> 0.079) independently predicted cardiovascular mortality (adjusted HR = 1.49, 95% CI:1.01–2.19, *p* = 0.045). When tested as a continuous variable, higher FAR levels were associated with a higher risk of cardiovascular (HR = 1.23, 95% CI: 1.04–1.47, *p* = 0.018) and all-cause mortality (HR = 1.12, 95%CI: 0.98–1.27, *p* = 0.090) in fully adjusted models. Subgroup analysis revealed that the association between higher FAR levels and increased cardiovascular mortality was significantly stronger in patients with triple-vessel disease (interaction *p* = 0.039). The associations between FAR and cardiovascular mortality remained robust in the Fine and Gray competing models (HR = 1.31, 95%CI: 1.13–1.52, *p* = 0.003).

**Conclusion:**

Higher FAR levels are associated with increased risks of cardiovascular and all-cause mortality in oldest-old patients undergoing PCI. These findings support the potential of FAR for risk stratification in geriatric cardiology.

**Supplementary Information:**

The online version contains supplementary material available at 10.1186/s12877-025-06111-4.

## Introduction

The prevalence of coronary artery disease (CAD) exhibits an age-dependent monotonic increase [1]. The aging population has contributed to a 60% increase in the absolute prevalence of CAD. This rising burden would mostly affect oldest-old patients (those aged ≥ 80 years) [2, 3]. Percutaneous coronary intervention (PCI) has become a routine therapeutic strategy for this geriatric population [4–6]. However, evidence-based risk stratification tools specifically tailored for post-PCI outcomes in oldest-old patients remain critically limited [7].

Emerging evidence suggests chronic inflammation as a key mediator in coronary atherosclerotic progression [8–10]. The fibrinogen-to-albumin ratio (FAR), integrating both pro-inflammatory (fibrinogen) and anti-inflammatory (albumin) pathways, has emerged as a novel inflammatory biomarker demonstrating strong associations with adverse cardiovascular outcomes in CAD populations [11, 12]. Fibrinogen, a plasma protein produced by the liver, plays a role in the processes of CAD, such as thrombosis formation and inflammatory response [11]. Conversely, albumin exerts protective effects via antioxidant, anti-inflammatory, and hemodilution properties. While previous investigations have established prognostic capacity of FAR in general cardiovascular contexts [12, 13], its clinical utility for predicting long-term mortality in oldest-old patients undergoing PCI remains unclear. This study therefore investigates the association between baseline FAR levels and long-term clinical outcomes in a cohort of oldest-old patients who underwent PCI.

## Methods

### Study population

**Fig. 1:**
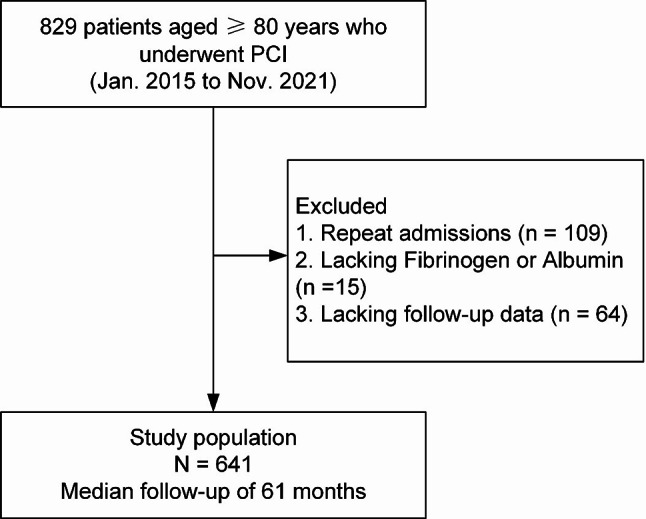
Patient flowchart of this study

This study was a single-center, retrospective, observational cohort study. CAD patients those aged 80 years or over who underwent PCI in Beijing Hospital from January 2015 to November 2021 were enrolled consecutively. Patients with any of the following conditions are considered ineligible for PCI and therefore were not enrolled in this study: severe liver disease (e.g., decompensated cirrhosis), acute critical infections (e.g., sepsis), malignancy with life expectancy < 1 year, major surgery or serious trauma within the preceding 3 months. The patient flowchart was shown in Fig. 1. Exclusion criteria included: (1) repeat admissions: for patients with multiple admissions, only the record from their first hospitalization was retained. (2) lacking results of fibrinogen or albumin; (3) lacking follow-up data. All participants signed the informed consent form. This study conformed to the Declaration of Helsinki (as revised in 2013) and was approved by the Ethics Committee of Beijing Hospital (No. 2022BJYYEC-085-01).

### Diagnosis of CAD

The diagnosis of CAD was based on coronary angiographic findings. The angiograms were obtained using standard techniques and evaluated by two cardiologists from multiple angles. The built-in QCA software of the Allura Xper FD20 Angiography System (Philips Healthcare, Netherlands) analyzed all targeted coronary lesions of the patients. CAD was defined as the presence of stenosis of 50% or higher in diameter in any of the coronary arteries or major branches.

### Clinical and laboratory data

Clinical parameters including demographic characteristics, risk factors, laboratory results, echocardiographic finding, and angiographic features were collected from the electronic medical records by trained clinicians. Fasting venous blood samples for biochemical parameters analysis were obtained within 24 h of admission before coronary angiography (CAG). Plasma fibrinogen concentrations (g/L) were measured on ACL-Top 700 (Werfen, USA) using Clauss fibrinogen assay. Serum albumin concentrations (g/L) were tested with an automated chemistry analyzer (HITACHI 7600, Japan). The fibrinogen-to-albumin ratio (FAR) was computed as the ratio of fibrinogen concentration divided by albumin level. All assays were performed by certified laboratory technicians who were blinded to clinical outcomes in the clinical laboratory of Beijing Hospital.

### Follow-up and outcomes

The primary endpoint was defined as cardiovascular mortality with all-cause mortality serving as the secondary endpoint. Mortality outcomes were determined through linkage to China’s National Mortality Surveillance System (NMSS), which legally records all registered deaths nationwide using participants’ unique national identification numbers, thereby ensuring near-complete ascertainment of mortality events. Cardiovascular deaths were classified according to the International Classification of Diseases, Tenth Revision (ICD-10) codes I00-I99. Follow-up for mortality events was completed by August 2024, ensuring complete endpoint ascertainment.

### Statistical analysis

Variables exceeding 20% missing data were excluded from analysis, with remaining missing values addressed via multiple imputation. Baseline characteristics were summarized as mean ± standard deviation (SD) for normally distributed continuous variables, median (interquartile range) for non-normally distributed variables, and frequencies (percentages) for categorical parameters. Patients were stratified based on the median of FAR (≤ 0.079 and > 0.079). Baseline characteristics between the lower FAR group (≤ 0.079) and higher FAR group (> 0.079) were compared using Student’s t-test, Wilcoxon rank-sum test, or Chi-square test as appropriate. Kaplan-Meier survival curves with log-rank tests evaluated mortality risk stratification by FAR levels. Unadjusted and adjusted hazard ratios (HRs) were derived from Cox proportional hazards regression models using four sequential adjustments: Model 1, Crude analysis; Model 2, adjusted for age and sex. Model 3, further adjustment for systolic blood pressure, previous myocardial infarction history and admission diagnosis. Model 4, additionally adjusted for white blood cell count, chronic kidney disease stage, uric acid, Ka, Na, and diuretic use. The effects of FAR on clinical outcomes were evaluated using both as continuous and categorical variables. Restricted cubic spline models with three knots were used to explore potential nonlinear associations between FAR and outcomes. Prespecified subgroup analyses evaluated effect modification by demographic/clinical factors using fully adjusted Model 4. Sensitivity analyses were performed. First, we excluded deaths within the first year to minimize the reverse causality. Second, we used the Fine and Gray subdistribution hazard model, an alternative approach to taking competing risk of mortality, to examine the associations between FAR and cardiovascular mortality. All statistical analyses were performed using R version 4.1.0 and GraphPad Prism version 8.0.1. Two-tailed p-values < 0.05 defined statistical significance.

## Results

### Baseline characteristics

A total of 641 patients were enrolled in the final analysis. Baseline characteristics are described in Table 1. The mean age was 82.8 ± 2.7 years and male patients accounted for 56.9%. Of these oldest-old patients, 71.8%, 43.2%, and 36.7% had hypertension, dyslipidemia, and diabetes mellitus (DM), respectively. 50.6% of the patients were diagnosed with stable angina and 60.7% had triple-vessel disease.

**Table 1:** Comparison of baseline characteristics of study population according to FAR median

Variables^a^	Overall	Lower FAR	Higher FAR	*p* value
	*N* = 641	*N* = 321	*N* = 320	
Sex, n (%)				0.062
Male	365 (56.9)	195 (60.7)	170 (53.1)	
Female	276 (43.1)	126 (39.3)	150 (46.9)	
Age, years	82.8 ± 2.7	82.6 ± 2.7	83.0 ± 2.7	0.02
BMI, kg/m^2	24.6 (3.35)	24.3 (3.25)	24.8 (3.44)	0.069
Hypertension, n (%)	460 (71.8)	230 (71.7)	230 (71.9)	1.000
DM, n (%)	235 (36.7)	105 (32.7)	130 (40.6)	0.046
Dyslipidemia, n (%)	277 (43.2)	142 (44.2)	135 (42.2)	0.657
Smoking history, n (%)	178 (27.8)	96 (29.9)	82 (25.6)	0.262
previous MI history, n (%)	125 (19.5)	60 (18.7)	65 (20.3)	0.676
SBP, mmHg	139 (124, 152)	140 (124, 151)	138 (123, 153)	0.813
DBP, mmHg	72.0 (64.0, 80.0)	73.0 (65.0, 80.0)	72.0 (63.0, 80.0)	0.194
HR, bpm	74.0 (65.0, 80.0)	72.5 (65.0, 80.0)	74.0 (65.0, 80.0)	0.609
Diagnosis, n (%)				0.001
STEMI	81 (12.7)	32 (10.0)	49 (15.4)	
NSTEMI	130 (20.3)	49 (15.3)	81 (25.4)	
Unstable Angina	105 (16.4)	57 (17.8)	48 (15.0)	
Stable Angina	323 (50.5)	182 (56.9)	141 (44.2)	
Lab results				
WBC, *10^9/L	6.37 (5.24, 7.77)	5.91 (5.05, 7.02)	6.79 (5.63, 8.19)	< 0.001
RBC, *10^9/L	4.05 (3.73, 4.38)	4.08 (3.77, 4.41)	4.03 (3.68, 4.34)	0.068
Hemoglobin, g/L	124 (114, 134)	126 (116, 135)	123 (112, 132)	0.005
Platelets, *10^12/L	188 (157, 226)	180 (149, 220)	196 (166, 241)	< 0.001
Neut, %	63.7 (57.1, 71.7)	62.2 (55.9, 69.6)	64.8 (58.1, 74.0)	< 0.001
FBG, mmol/L	5.70 (5.10, 6.80)	5.60 (5.00, 6.60)	5.80 (5.10, 7.30)	0.031
albumin, g/L	39.0 (37.0, 41.0)	40.0 (38.0, 42.0)	38.0 (36.0, 40.0)	< 0.001
Uric Acid, mmol/L	348 (277, 421)	337 (279, 407)	360 (273, 434)	0.05
Ka, mmol/L	4.10 (3.80, 4.30)	4.10 (3.90, 4.30)	4.10 (3.80, 4.30)	0.985
Na, mmol/L	141 (139, 142)	141 (139, 142)	140 (138, 142)	0.224
eGFR, mL/min/1.73 m^2	52.5 (42.3, 65.7)	55.4 (46.3, 66.2)	49.6 (39.0, 64.7)	< 0.001
CKD stage, n (%)				0.026
Stage 1	26 (4.06)	13 (4.06)	13 (4.06)	
Stage 2	193 (30.2)	109 (34.1)	84 (26.2)	
Stage 3	374 (58.4)	183 (57.2)	191 (59.7)	
Stage 4	39 (6.09)	14 (4.38)	25 (7.81)	
Stage 5	8 (1.25)	1 (0.31)	7 (2.19)	
TC, mmol/L	3.65 (3.18, 4.40)	3.58 (3.11, 4.23)	3.74 (3.24, 4.56)	0.072
TG, mmol/L	1.11 (0.80, 1.50)	1.06 (0.77, 1.44)	1.16 (0.83, 1.59)	0.068
HDL-C, mmol/L	1.04 (0.89, 1.23)	1.07 (0.92, 1.26)	1.02 (0.87, 1.19)	0.008
LDL-C, mmol/L	2.10 (1.66, 2.70)	2.01 (1.62, 2.55)	2.20 (1.70, 2.90)	0.004
Hb1Ac, %	6.50 (6.00, 7.47)	6.30 (6.00, 7.25)	6.60 (6.00, 7.70)	0.101
BNP, pg/ml	203 (88.0, 532)	163 (75.8, 426)	243 (101, 661)	0.002
FIB, g/L	3.11 (2.69, 3.63)	2.69 (2.38, 2.93)	3.63 (3.28, 4.06)	< 0.001
LVEF < 40%, n (%)	64 (11.2)	26 (9.09)	38 (13.3)	0.14
LVEDD, mm	46 (43, 49)	46 (43, 48)	47 (43, 50)	0.559
LADD, mm	37 (34.0, 41)	36 (33, 41)	37 (34, 42)	0.046
Angiographic data, n (%)				0.167
Single-vessel disease	85 (13.3)	42 (13.1)	43 (13.4)	
Double-vessel disease	167 (26.1)	94 (29.3)	73 (22.8)	
Triple-vessel disease	389 (60.7)	185 (57.6)	204 (63.7)	
Medication, n (%)				
β-blockers	423 (67.5)	215 (67.8)	208 (67.1)	0.913
RASi	329 (52.6)	169 (53.5)	160 (51.6)	0.698
CCB	255 (40.7)	136 (42.9)	119 (38.4)	0.285
Statins	621 (99.2)	313 (99.1)	308 (99.4)	1.000
Diuretics	114 (18.2)	41 (12.9)	73 (23.5)	0.001
Spirolactone	76 (12.1)	27 (8.52)	49 (15.8)	0.008
FAR	0.079(0.067–0.093)	0.067 (0.060–0.073)	0.093(0.086–0.109)	< 0.001

Abbreviations: BMI, body mass index; DM, diabetes mellitus; SBP, Systolic blood pressure; DBP, Diastolic blood pressure; HR, heart rate; STEMI, ST segment elevation myocardial infarction; NSTEMI, non-ST segment elevation myocardial infarction; WBC, white blood cell; RBC, red blood cells; FBG, fasting blood glucose; eGFR, estimated glomerular filtration rate; CKD, chronic kidney disease; TC, total cholesterol; TG, triglycerides; HDL-C, high-density lipoprotein cholesterol; LDL-C, low-density lipoprotein cholesterol; BNP, Brain Natriuretic Peptide; FAR, Fibrinogen-to-Albumin Ratio; LVEF, left ventricular ejection fraction; LVEDD, left ventricular end-diastolic dimension; LADD, left atrial diastolic dimension; RASi, renin-angiotensin-system inhibitors; CCB, Calcium channel blocker; FAR, Fibrinogen-to-albumin ratio

^a^Data are mean ± SD, median (interquartile range) for continuous variables, or n (%) for categorical variables

The mean of FAR was 0.082 ± 0.023 with the median of 0.079 (0.067–0.093). Stratification by median FAR revealed significant intergroup differences. Compared to the lower FAR group (≤ 0.079), patients in the higher FAR group (> 0.079) tended to be older, to have a higher proportion of DM and more probably to be diagnosed with myocardial infarction. Additionally, patients in the higher FAR group had a higher white blood cell counts, higher platelet counts, lower hemoglobin, higher low-density lipoprotein cholesterol (LDL-C), lower estimated glomerular filtration rate (eGFR) and higher brain natriuretic peptide (BNP).

### Clinical outcomes

The median follow-up time was 61 months. During follow-up, 237 (37%) mortality events were recorded, including 124 cardiovascular-specific deaths (19.3%). The 1-year and 5-year mortality rates were 9.3% and 27.4%, respectively.

### FAR levels and clinical outcomes

**Fig. 2:**
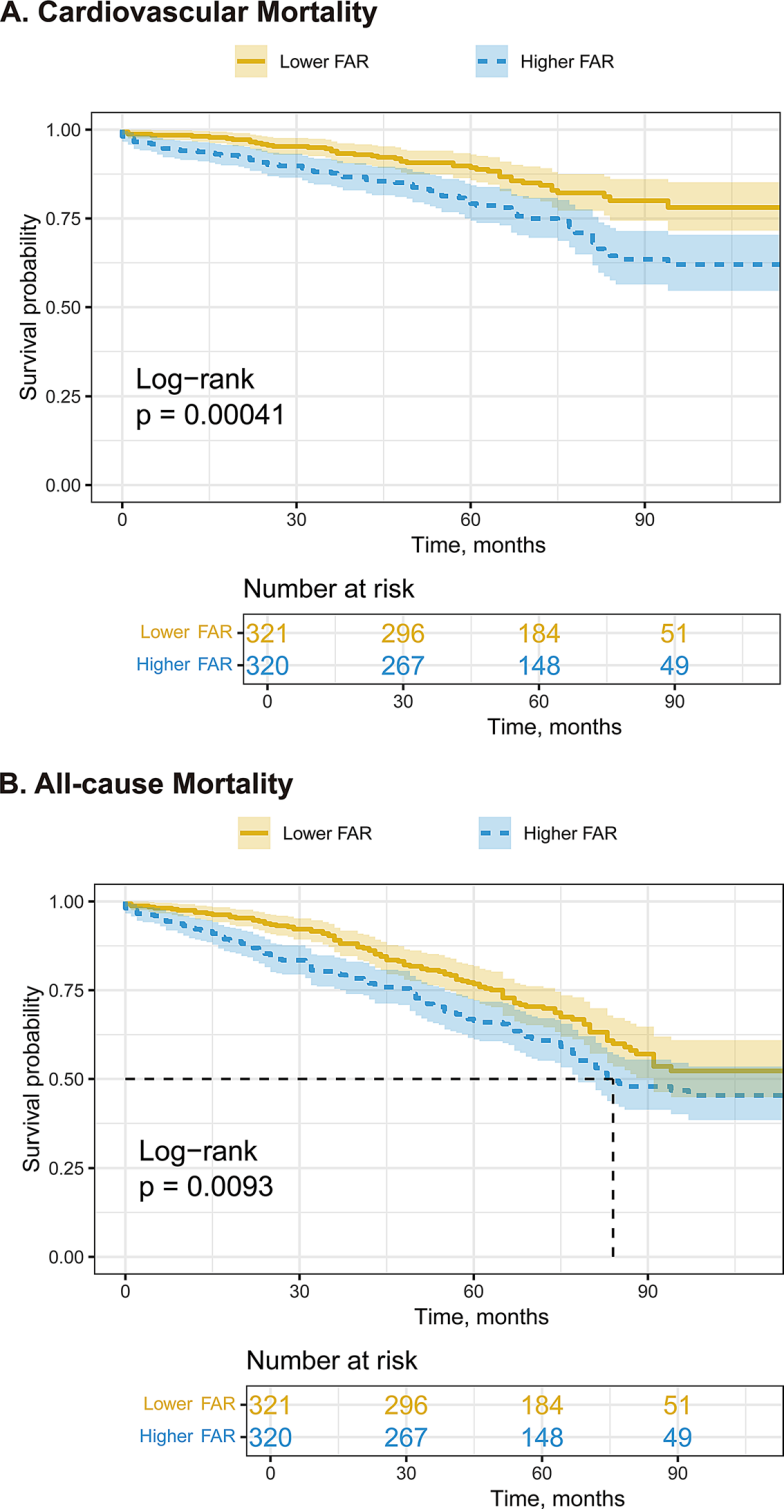
Kaplan-Meier curves of cardiovascular mortality stratified by median of FAR. FAR, fibrinogen-to-albumin ratio

**Fig. 3:**
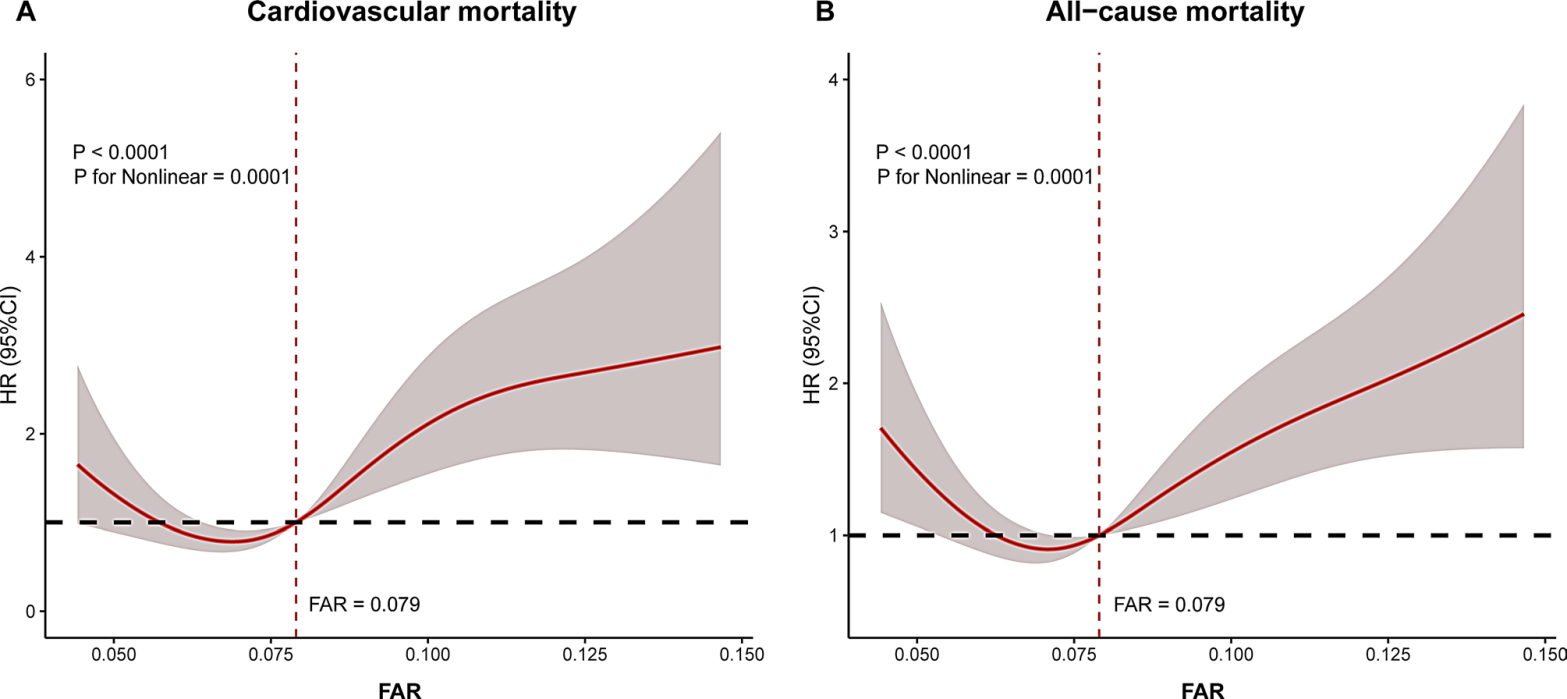
The association of FAR with cardiovascular mortality (**A**) and all-cause mortality (restricted cubic spline analysis with three knots adjusted for age and sex). CI, confidence interval; FAR, Fibrinogen-to-Albumin Ratio; HR, Hazard ratio

Kaplan-Meier analysis demonstrated significantly increased risks of cardiovascular mortality (log-rank *p* < 0.001) and all-cause mortality (log-rank *p* < 0.001) in patients with higher FAR levels (Fig. 2). Restricted cubic spline analysis revealed J-shaped associations between FAR and mortality outcomes, with nonlinear trends observed for both cardiovascular mortality (*p* for overall < 0.0001, *p* for nonlinear = 0.0001) and all-cause (*p* for overall < 0.0001, *p* for nonlinear = 0.0001) (Fig. 3). FAR values above 0.079 were associated with an increased risk of mortality.

Using Cox proportional hazards models, we further examined the association between risk of clinical outcomes and FAR, as shown in Table 2. In the crude model of univariate Cox regression analysis, higher FAR was associated with a higher rate of cardiovascular mortality (HR = 1.91, 95% CI: 1.32–2.76, *p* < 0.001) compared to lower FAR group. After full adjustment for covariates (Model 4), the cardiovascular mortality in higher FAR group remained significantly higher than the lower FAR group (HR = 1.49, 95% CI: 1.01–2.29, *p* = 0.045). When tested as a continuous variable, per SD increase predicted 145% higher risk (HR = 1.23, 95% CI: 1.04–1.47, *p* = 0.018).

**Table 2:** Associations of FAR with cardiovascular mortality and all-cause mortality according from the four models

	Model 1			Model 2			Model 3			Model 4	
	**HR (95%CI)**	***P*** **-value**		**HR (95%CI)**	***P*** **-value**		**HR (95%CI)**	***P*** **-value**		**HR (95%CI)**	***P*** **-value**
**Cardiovascular mortality**											
Per SD	1.36 (1.17, 1.59)	< 0.001		1.36 (1.16, 1.59)	< 0.001		1.25 (1.06, 1.47)	0.007		1.23 (1.04, 1.47)	0.018
lower FAR	Reference			Reference			Reference			Reference	
Higher FAR	1.91 (1.32, 2.76)	< 0.001		1.83 (1.26, 2.56)	0.002		1.62 (1.11, 2.36)	0.012		1.49 (1.01, 2.19)	0.045
**All-cause mortality**											
Per SD	1.22(1.08, 1.37)	0.002		1.22(1.07, 1.38)	0.002		1.43(1.01, 1.29)	0.037		1.12 (0.98, 1.27)	0.090
lower FAR	Reference			Reference			Reference			Reference	
Higher FAR	1.40 (1.08, 1.81)	0.010		1.36 (1.05, 1.76)	0.022		1.25 (0.96, 1.63)	0.097		1.13 (0.86, 1.49)	0.361

Model 1: crude risk;

Model 2: adjusted age and sex;

Model 3: Model 2 + SBP, previous MI history, diagnosis;

Model 4: Model 3 + WBC, CKD stage, Uric Acid, Ka, Na, use of diuretics

Abbreviations: CI, Confidence interval; FAR, Fibrinogen-to-albumin ratio; HR, Hazard ratio; SBP, systolic blood pressure; MI, myocardial infarction; WBC, white blood cells; CKD, chronic kidney disease

For patients in higher FAR group, the unadjusted HR for all-cause mortality was 1.40 (95% CI: 1.08–1.81, *p =* 0.010) compared to individuals in lower FAR group. In adjusted model 2, individuals in higher FAR group had higher risk of all-cause mortality compared to individuals in lower FAR group (adjusted HR = 1.36, 95% CI: 1.05–1.76). These associations were attenuated after additional adjustment for CAD risk factors (Model 3, adjusted HR: 1.25, 95%CI: 0.96–1.63, *p* = 0.097; Model 4, adjusted HR: 1.13, 95%CI: 0.86–1.49, *p* = 0.361). When tested as a continuous variable, higher FAR was associated with a higher risk of all-cause mortality in both unadjusted and fully adjusted models (Model 4, HR = 1.12, 95%CI: 0.98–1.27, *p* = 0.090).

### Subgroup analysis

**Fig. 4:**
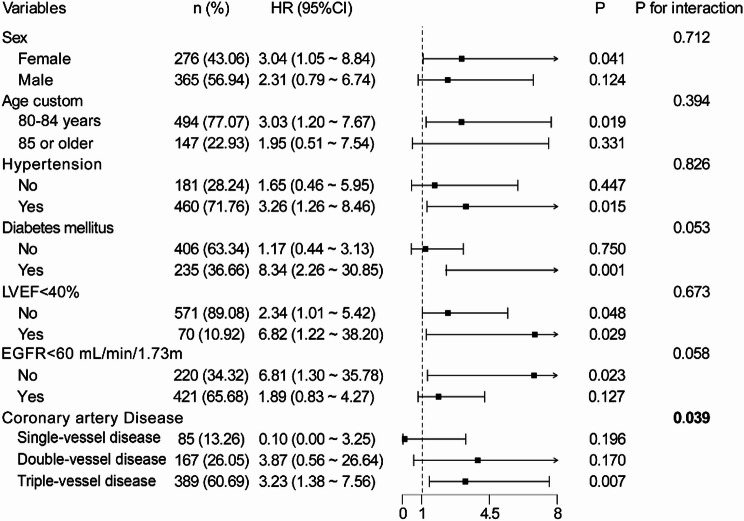
Subgroup analysis of relationship between FAR levels and cardiovascular mortality. CI, confidence interval; EGFR, estimated glomerular filtration rate; FAR, Fibrinogen-to-Albumin Ratio; HR, Hazard ratio. LVEF, left ventricular ejection fraction

In the subgroup analysis, a higher FAR was consistently associated with increased cardiovascular mortality across various subgroups, including age, sex, hypertension, DM, left ventricular ejection fraction (LVEF) and eGFR (Fig. 4). No significant interactions were observed between FAR and these variables (all *p* values for interaction > 0.05). However, a significant interaction was found between FAR and the severity of CAD (*p* value for the interaction = 0.039). Specifically, in the triple-vessel disease group, a higher FAR was significantly associated with increased cardiovascular mortality.

### Sensitivity analyses

To minimize the reverse causation, we excluded 36 patients who died within 1 years of follow-up. The Kaplan-Meier analysis (Supplement Fig. 1) demonstrated significantly increased risks of cardiovascular mortality (log-rank *p* = 0.009) in patients with higher FAR levels. These associations were not significant for all-cause mortality. The associations between FAR and cardiovascular mortality remained robust in the Fine and Gray competing models (HR = 1.31, 95%CI: 1.13–1.52, *p* = 0.003) (Supplement Fig. 2).

## Discussion

In this retrospective study, we investigated the association of FAR levels with long-term outcomes in oldest-old patients (aged ≥ 80 years) with CAD who underwent PCI. Our results showed the 1-year mortality rate among these patients was 9.3% and the 5-year mortality rate was 27.4%. Higher FAR levels were associated with an increased risk of both cardiovascular and all-cause mortality. According to the restricted cubic spline analysis, the relationship between FAR and mortality followed a J-shaped pattern, with FAR values above 0.079 linked to a higher risk of mortality. After adjusting for clinical variables, higher FAR remained independently associated with cardiovascular mortality. These results suggest that FAR, as a novel inflammatory biomarker, may serve as a valuable prognostic tool for risk stratification in elderly patients undergoing PCI.

The baseline characteristics of our study population indicated that patients with higher FAR levels tended to be older, had a higher prevalence of DM, and were more likely to have a history of myocardial infarction. Notably, the higher FAR group demonstrated persistently elevated leukocyte counts, lower hemoglobin levels and reduced eGFR. These findings align with FAR’s dual role as a composite biomarker of systemic inflammation (reflected by leukocytosis and hypoalbuminemia) and metabolic stress (evidenced by anemia and renal dysfunction), consistent with the ‘inflammaging’ paradigm observed in advanced age [14]. Our results are consistent with previous studies highlighting the role of inflammation and metabolic dysregulation in the progression of CAD, particularly among elderly individuals [15, 16].

Atherosclerosis is a lipid-driven inflammatory disease of the arterial intima, where the balance of pro-inflammatory and inflammation-resolving mechanisms determines clinical outcomes [8, 17, 18]. Fibrinogen, primarily synthesized in the liver, functions not only as a prothrombotic factor but also as a pro-inflammatory glycoprotein [19–21]. Elevated fibrinogen levels are recognized as a hallmark of chronic inflammatory states, particularly in conditions such as DM and atherosclerotic disease [8, 22]. In contrast, albumin exerts antioxidant and anti-inflammatory effects [23, 24]. Epidemiological evidence indicates that low serum albumin levels are linked to incident ischemic heart disease, heart failure, atrial fibrillation, stroke and venous thromboembolism, independent of traditional risk factors, body mass index and inflammation [25, 26]. Moreover, hypoalbuminemia has also emerged as an independent prognostic indicator in many cardiovascular diseases [25].

The FAR integrates two opposing biological pathways: one driving thrombosis and inflammation (fibrinogen) and the other exerting anti-inflammatory and protective effects (albumin). A higher FAR may indicate a systemic imbalance favoring a proinflammatory state, which is particularly detrimental in elderly patients with diminished physiological reserve. Recent research by Lan et al. [11] supports this hypothesis, demonstrating that fibrinogen directly enhances macrophage activation and cytokine release in atherosclerotic plaques. Our findings that higher FAR correlated with lower hemoglobin, higher BNP, and reduced eGFR suggest that this ratio may also encapsulate broader systemic dysfunctions, such as anemia, heart failure, and renal impairment, all of which are prevalent in the elderly and contribute to a poorer prognosis.

Our findings revealed a J-shaped relationship between FAR and mortality, with a critical threshold of 0.079. Beyond this point, the risk of cardiovascular and all-cause mortality increased significantly. This pattern aligns with recent studies that emphasize the nonlinear association between inflammatory markers and adverse outcomes in CAD. Fang et al. [13] observed a similar trend in patients with myocardial infarction with non-obstructive coronary arteries, demonstrating FAR’s prognostic value independent of conventional risk factors. Similarly, Li et al. [12] established FAR’s superior predictive capacity compared to isolated fibrinogen or albumin measurements in three-vessel disease cohorts, consistent with our subgroup analysis revealing enhanced prognostic utility in triple-vessel disease patients.

The persistent association between higher FAR and cardiovascular mortality following multivariable adjustment suggests its dual role as a composite biomarker of systemic inflammation and metabolic dysregulation. However, the attenuation of the association with all-cause mortality in fully adjusted models implies competing risks from non-cardiovascular comorbidities—particularly frailty and renal dysfunction—in this octogenarian population [3]. This aligns with recent studies emphasizing the multifactorial etiology of mortality in advanced age, where cumulative comorbidity burden often supersedes traditional cardiovascular risk predictors [27]. Revascularization technique and adjunctive medical therapy can substantially influence long-term outcome. In the future, we will collect data on frailty, treatment adherence and revascularization completeness to fully disentangle FAR’s prognostic value from potential therapeutic confounders.

Subgroup analyses further confirmed the robustness of our findings, demonstrating consistent association between higher FAR and cardiovascular mortality across subgroups, including those stratified by age, sex, hypertension, DM, LVEF, and eGFR. Notably, interaction testing revealed significant effect modification by coronary disease severity, with substantially amplified FAR-mortality associations in triple-vessel disease patients, indicating enhanced prognostic utility in advanced CAD phenotypes.

The clinical implications of FAR-guided risk stratification warrant further exploration. High-FAR patients may benefit from intensified anti-inflammatory therapies, personalized pharmacotherapy targeting metabolic dysregulation, and multidisciplinary comorbidity management. Future randomized controlled trials evaluating FAR-driven treatment algorithms (e.g., colchicine in high-FAR subgroups) are needed to validate these approaches [28].

This study has several limitations. First, this was a single-center, retrospective study with several inherent limitations, including selection bias. We included consecutive patients during the study period, which might have helped to minimize the bias. Second, although patients with absolute or relative PCI contraindications—including severe liver disease, sepsis, advanced malignancy, recent major surgery, or trauma were excluded in our study, residual confounding by subclinical inflammation or nutritional disturbances might persist. Residual confounding (e.g., unmeasured frailty indices or socioeconomic factors) may persist despite multivariable adjustments. Third, the exclusion of patients with missing data could introduce selection bias. Forth, our study is the unavailability of socioeconomic status indicators, such as income, education, and occupation, which are known to influence cardiovascular risk and long-term outcomes. Finally, the mechanisms linking FAR to mortality remain speculative; future studies should explore causal pathways using multi-omics approaches or interventional designs.

## Conclusion

Our study demonstrates that higher FAR levels are associated with increased risks of both cardiovascular and all-cause mortality in oldest-old patients undergoing PCI. These findings indicate that FAR may serve as a valuable prognostic biomarker for risk stratification in this vulnerable population. Future studies should explore the potential utility of FAR for guiding therapeutic interventions and improving outcomes in elderly patients with CAD.

## Electronic supplementary material

Below is the link to the electronic supplementary material.

Supplementary Material 1

## Data Availability

The datasets used and analyzed during the current study are available from the corresponding author on reasonable request.

## Abbreviations

FAR: FARibrinogen-to-albumin ratio
CAD: Coronary artery disease
PCI: Percutaneous coronary intervention
NMSS: National Mortality Surveillance Systems
SD: Standard deviation
DM: Diabetes mellitus
LDL: C low-density lipoprotein cholesterol
eGFR: Lower estimated glomerular filtration rate
BNP: Brain natriuretic peptide
LVEF: Left ventricular ejection fraction
HR: Hazard ratio
CI: Confidence interval
