# Supplementary material for: Fibrinogen-to-albumin ratio and long-term mortality in oldest-old patients undergoing percutaneous coronary intervention
Source: BMC Geriatr. 2025 Jul 2;25:460. doi: 10.1186/s12877-025-06111-4 (PMC12219949; doi:10.1186/s12877-025-06111-4)

Supplement Figure 1. Sensitivity analyses of association between FAR and outconmes (exclude individuals died within 1 years of follow-up).


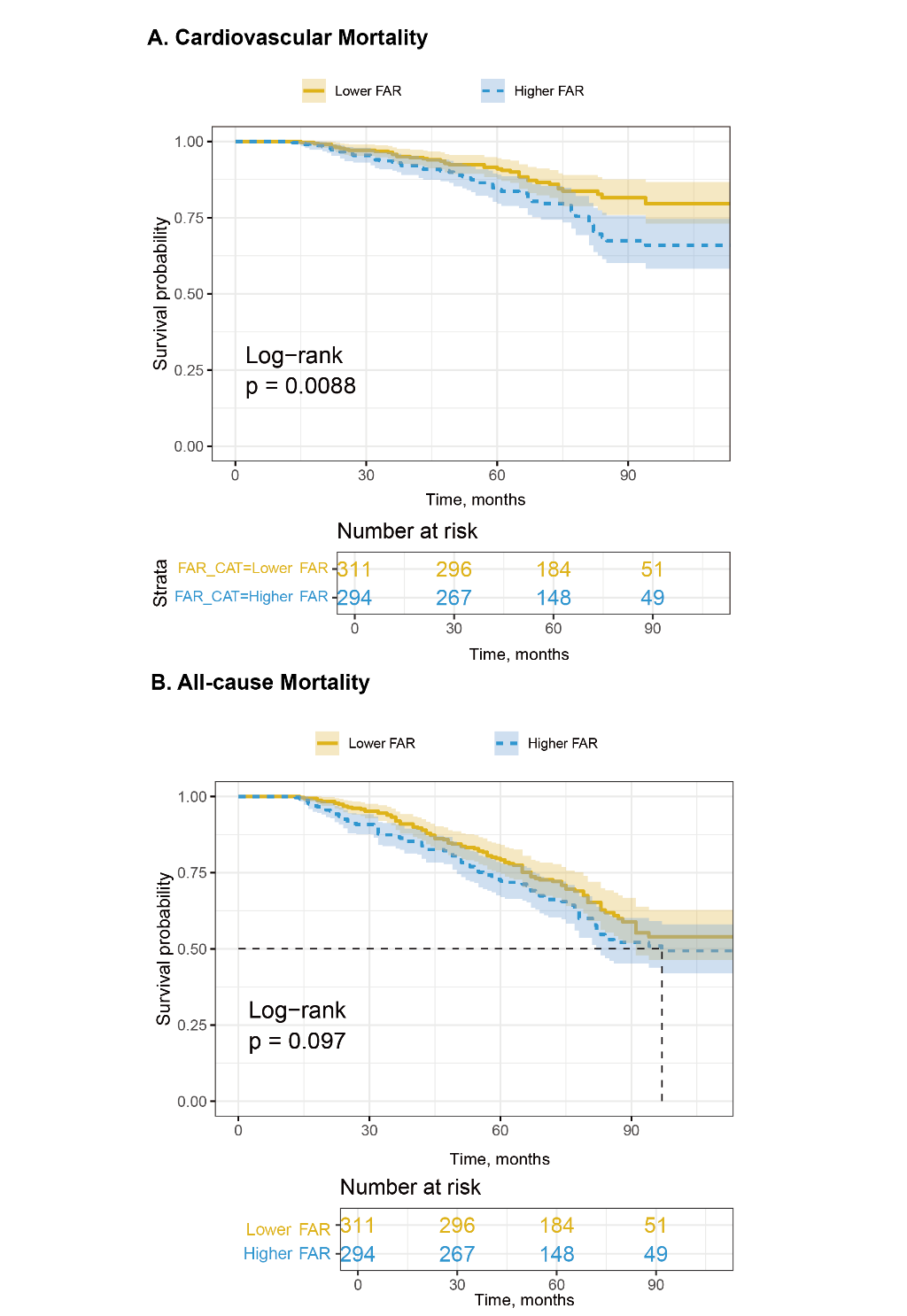


Supplement Figure 2. Association between FAR and risk of outcomes using Fine & Gray models for competing risk.


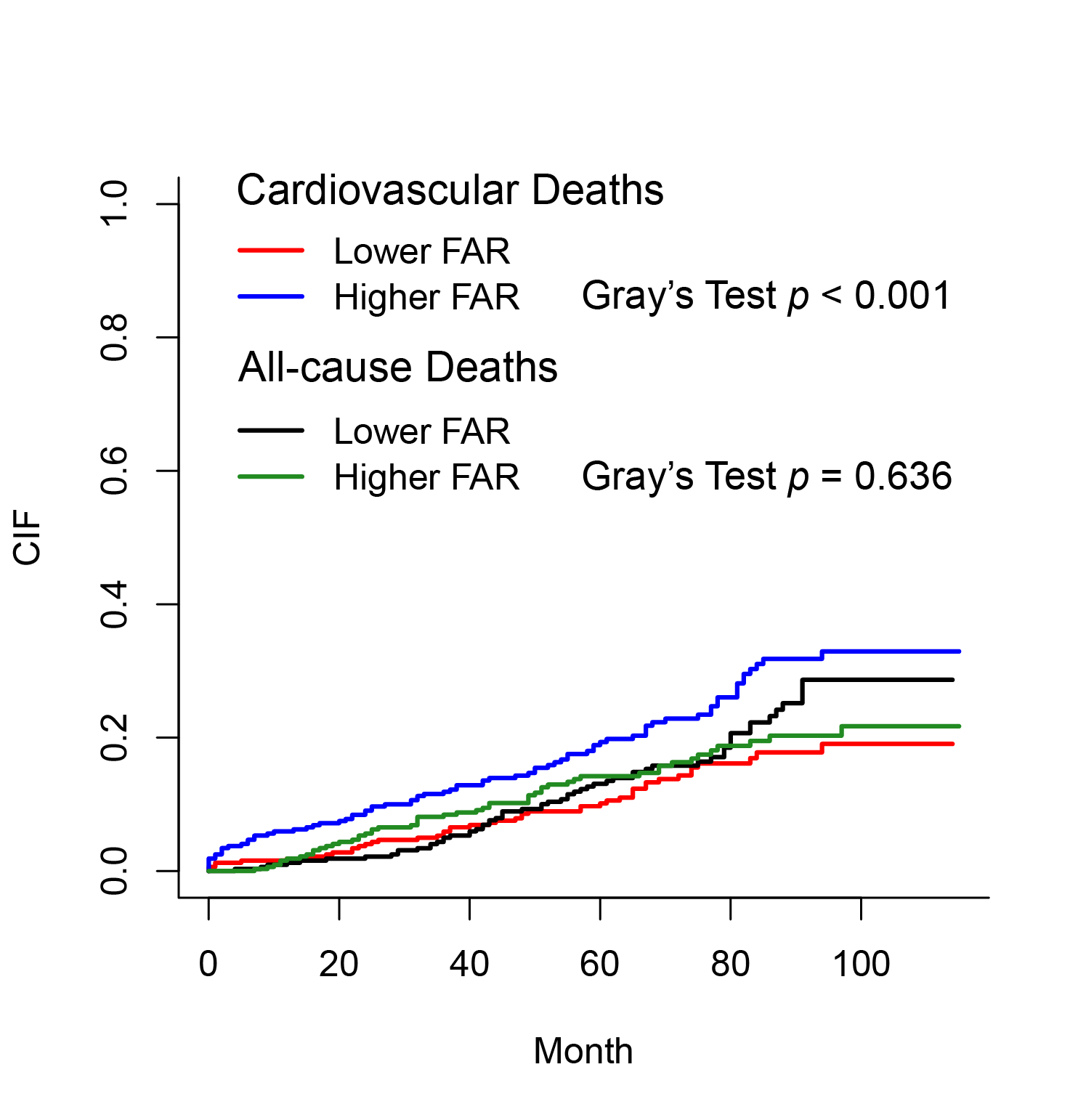

Supplement: Supplementary file 1 — Supplementary Material 1 [file 12877_2025_6111_MOESM1_ESM.docx]
